# Utility of Pulmonary Angiography by 128-Slice Computed Tomographic Scanner in Diagnosis of Tetralogy of Fallot Cases

**DOI:** 10.1155/2024/3543906

**Published:** 2024-05-09

**Authors:** Abhishek Dwivedi, Ankur Sharma, Rachit Sharma, Prateek Awasthi, Satveer Singh Choudhary

**Affiliations:** ^1^FH Medical College, Agra, India; ^2^Department of Radiology, Military Hospital, Jaipur, Rajasthan, India; ^3^OIC Department of Radiology, Military Hospital, Agra, India; ^4^Department of Radio Diagnosis, BHDC and Army College of Medical Sciences, Delhi, India

## Abstract

Tetralogy of Fallot (TOF) is a significant cause of cyanotic congenital heart disease (CHD) encountered in childhood with few cases manifesting in adulthood. It has four classical features (ventricular septal defect, overriding of aorta, hypertrophy of right ventricular hypertrophy, and right ventricular outflow tract obstruction), but the clinical presentation and course can be variable. Due to various anatomical variations and complex anatomy, presurgical planning and postoperative follow-up by pulmonary computed tomographic angiography (CTA) have a very important role. With continued technological advances and the availability of 128-slice computed tomographic (CT) scans, they now play an important role in TOF preoperative evaluation and workup, assisting by minimizing routine invasive digital subtraction catheter angiography. The fast scan of a 128-slice CTA with very sensitive detectors is a very useful modality for studying the complex anatomy and variations as well as its utilization for postoperative management. In this article, we report four cases of TOF where we used a 128-slice scan for performing pulmonary angiography (Optima 660, GE 128, 2180 Premier Row, Orlando, FL 32809, U.S.A.) for preoperative diagnosis and management of three cases and work up for revision surgery for an already operated case with a nonfunctional modified Blalock-Taussig shunt with additional lung parenchymal findings simultaneously. This study will explain the advantageous role of the 128-slice CT scanner over the lesser-slice CT scanners with the ability of pulmonary CTA to facilitate accurate diagnosis and postoperative management.

## 1. Introduction

The diagnosis of Tetralogy of Fallot (TOF) can be done in the antenatal period by fetal ultrasound imaging. The postnatal first diagnosis is done by echocardiography with pulmonary angiography. Here, we used a 128-slice computed tomography (CT) scan (Optima 660, GE 128, 2180 Premier Row, Orlando, FL 32809, U.S.A.) with intravenous nonionic contrast media injectable iohexol 350 mg I/ml with the help of a pressure injector at a flow rate of 5 ml/sec with a dose of 1.5 ml/kg body weight (1 ml/kg for children less than 5 years and 1.5 ml/kg for children greater than 5 years). The flow rate for contrast is 5 ml/sec, followed by the saline chaser at a flow rate of 3 ml/sec (this is low for infants with a flow rate of 3 ml/sec for contrast and saline). Here, we performed pulmonary CT angiography (CTA) with a protocol of 1.25 mm slice thickness, acquired in helical scan mode, taken from the thoracic inlet to the diaphragm with a pitch of 1.375 with a speed of 155.00 mm per rotation and a rotational time of 0.5 sec after IV bolus of contrast and bolus tracking at the marker until the Hounsfield units reach 100 with marker at the main pulmonary artery. The image reconstruction was done at 1.25 mm intervals at 120 kV (100 mA for children and 200 mA for adults). After the acquisition of noncontrast images and the contrast, a study is acquired, followed by acquired image reformation in all three planes using a dual-head injector (Stellant D Dual Syringe CT Injection System; Medrad, Warrendale, PA, USA). In this study, we covered four patients. All of them have been diagnosed with TOF both clinically and by echocardiography. Three of them were evaluated for surgery; however, one of the cases was operated on 10 years back for a modified Blalock-Taussig shunt and was again planned for rectification surgery with a suspicion of palliative shunt blockage. One case had an additional atrial septal defect, making this a variant known as the Pentalogy of Fallots.

### 1.1. Case 1

**Figure 1:**
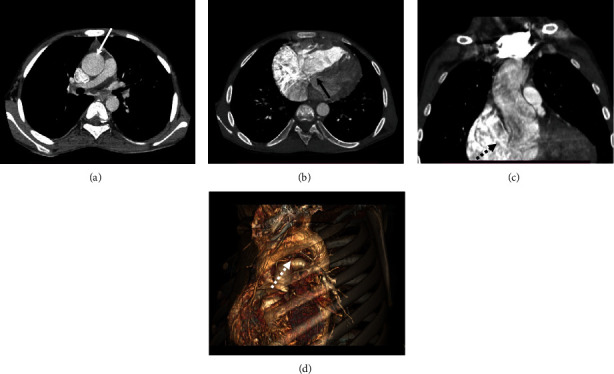
A 15-year-old male with Tetralogy of Fallot. (a) Dilated ascending aorta (white arrow) in axial section. (b) Large ventricular septal defect (black arrow) in axial section. (c) Overriding of aorta with ventricular septal defect (black dotted arrow) in coronal section. (d) Volume-rendered image with left-sided aortic arch (dotted white arrow).

A 15-year-old male with chief complaints of breathlessness and cyanosis manifested in childhood at the age of five years. Echocardiography shows a very small pulmonary artery with infundibular pulmonary stenosis, a ventricular septal defect, and a dilated right chamber of the heart. Pulmonary CTA shows the arch of the aorta is on the left side, the aorta is overriding, and dilated ascending aorta (Figure 1(a)); descending aorta is normal in caliber. A large ventricular septal defect was noted in the subaortic region (Figures 1(b) and 1(c)), with dilated right-side heart chambers. The pulmonary main trunk and arteries are mildly smaller. The arch of the aorta is left-sided (Figure 1(d)).

### 1.2. Case 2

**Figure 2:**
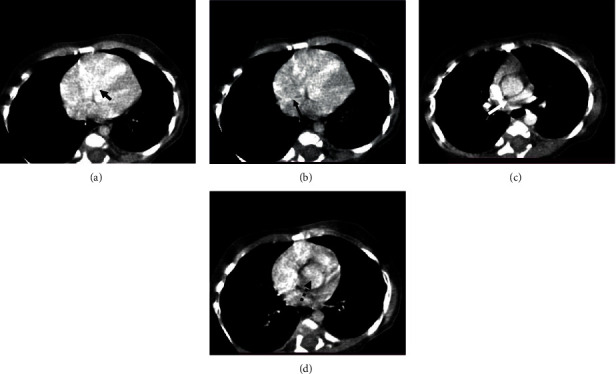
A 9-month-old male with Tetralogy of Fallot with axial sections. (a) Large ventricular septal defect (short black arrow). (b) Small atrial septal defect (long black arrow). (c) Hypoplastic right pulmonary artery (white arrow) and absent left pulmonary artery. (d) Overriding of aorta with ventricular septal defect (black dotted arrow).

A nine-month-old male infant with a normal birth history came with chief complaints of excessive crying, cyanosis, and frequent diarrhea. The echocardiography findings are situs solitus, levocardia, dilated both right heart chambers, ostium secundum type atrial septal defect with bidirectional shunt, nonrestrictive subaortic type ventricular septal defect with bidirectional shunt, and severe infundibular valvular and supravalvular type pulmonary stenosis. The main pulmonary artery is small, the left pulmonary artery is not seen, and the right pulmonary artery is also small. On pulmonary CTA findings, a subaortic ventricular septal defect (Figure 2(a)) is seen. A small radiologically visible atrial septal defect is seen measuring 2 mm (Figure 2(b)). The normal pulmonary trunk is seen for a length of 21 mm and continues as the right main pulmonary artery (Figure 2(c)); the left pulmonary artery is not visualized. The aorta is overriding (Figure 2(d)). Multiple left-side aortopulmonary collaterals are seen, originating from the descending aorta and supplying the left lung.

### 1.3. Case 3

**Figure 3:**
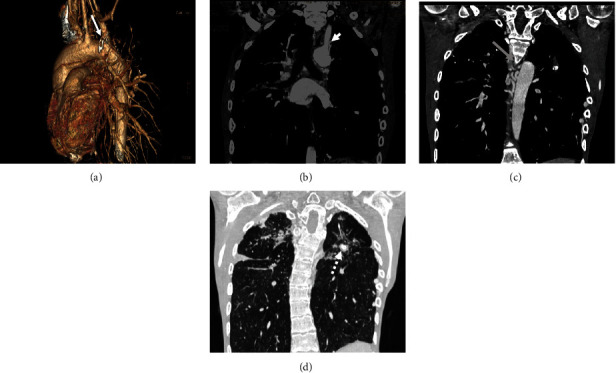
An 18-year-old male with Tetralogy of Fallot. (a) Modified Blalock-Taussig shunt (white arrow) in volume-rendered image. (b) Blocked modified Blalock-Taussig shunt (white arrowhead) in maximum intensity projection images. (c) Multiple aortopulmonary collateral arteries (black arrow), few collaterals at the inferior margin of lower ribs in coronal images. (d) Tubercular changes with calcified and cavities in bilateral upper lobes of the lung (white dotted arrow) in lung window coronal section.

An 18-year-old male diagnosed with TOF was operated on 8 years back with a modified Blalock-Taussig shunt and is presently presenting with complaints of breathlessness, cyanosis, and hemoptysis. He had a history of pulmonary tuberculosis, for which he completed antitubercular therapy 5 years back. There is a clinical suspicion of a shunt block. Pulmonary CTA findings show a visible shunt connecting the left pulmonary artery to the left subclavian artery (Figures 3(a) and 3(b)) with no flow and hypodense contents in the lumen, suggestive of a complete block. Major aortopulmonary collaterals (Figure 3(c)) are seen originating from the posterior and right lateral borders of the descending aorta and the anterior aspect of the lower thoracic aorta, causing collateralization along the inner and inferior margins of bilateral upper ribs with scalloping. On the reformed lung window (Figure 3(d)), noncontrast images show fibrotic areas with calcification and cavities are noted in the right upper lobe, right middle lobe, left upper lobe, and lateral and posterior basal segments of the right lower lobe, suggesting a recurrence of tubercular lesions. There is also an overriding of the aorta with fusiform dilatation of the ascending aorta. A large perimembranous ventricular septal defect is also seen in this case. The pulmonary trunk and the right pulmonary artery are normal, with a small-caliber left pulmonary artery along with right heart enlargement.

### 1.4. Case 4

**Figure 4:**
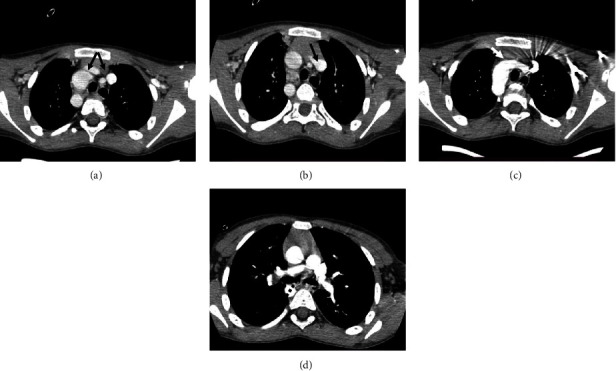
A 6-year-old male with Tetralogy of Fallot with axial sections. (a) Bilateral innominate veins (black double arrow). (b) Left-sided superior vena cava (black arrow). (c) Right-sided aortic arch (white arrow). (d) Bilateral hypoplastic but present pulmonary arteries (dotted black arrow).

A six-year-old male with chief complaints of breathlessness and cyanosis manifested in childhood at the age of three years. Echocardiography findings are situs solitus, levocardia, right side aortic arch, left side superior vena cava, and normal trunk with the left pulmonary artery not visualized. Subaortic type ventricular septal defect with overriding of aorta. The pulmonary CTA shows both innominate veins draining into the left side and the superior vena cava (Figures 4(a) and 4(b)), which is draining into the right atrium after crossing to the right side. The aorta is seen originating from the midline of the left ventricle, with a septal defect of 5 mm in the subaortic region of the ventricular septum. The arch of the aorta is seen on the right side (Figure 4(c)), with the thoracic aorta also seen on the right side of the midline and the abdominal aorta on the midline. The pulmonary trunk is seen originating from the right ventricle, the main pulmonary artery measures 13 mm in diameter, and the left pulmonary artery is mildly hypoplastic, as seen in its entire course (Figure 4(d)).

## 2. Discussion

The prevalence of TOF in India is 1.13 per 1000 live births, and this makes this disease a very important cardiac anomaly to manage [1]. The imaging and diagnosis of TOF can be done using various techniques. The antenatal diagnosis can be done by ultrasound and color Doppler studies [2]; the first postnatal diagnosis is done by echocardiography. The ultrasound and echocardiography modalities are easily available modalities. However, as operator-based variation with their two-dimensional acquisition and inability to comment about the coronary arteries, early aortopulmonary collateral arteries, and less delineation of the hypoplastic pulmonary artery and its branches, the inability to comment on associated extracardiac anomalies makes these modalities underutilized for better diagnosis and further management. Pulmonary computed tomography angiography (CTA) has a unique role in the diagnosis as it shows more three-dimensional (3D) pictures as well as better visualization of associated findings that can be missed on conventional imaging techniques. The edge of this machine allows for better visualization of the cardiac and coronary anatomy [3].

Tetralogy of Fallot (TOF) is a cyanotic congenital heart disease (CHD) with the cardiac manifestations of four classical features (ventricular septal defect, overriding of aorta, hypertrophy of right ventricular hypertrophy, and right ventricular outflow tract obstruction) [4], but the clinical presentation and anatomical variations lead to the need for additional information which can be missed by conventional imaging techniques. The mortality of the TOF is also significant; hence, proper diagnosis and management are significant [5]. Echocardiography is the main modality for postnatal diagnosis. However, due to various anatomical variations and complex anatomy, presurgical planning, and postoperative follow-up, pulmonary CTA has a very important role. With continued technological advances, powerful and more detectors along with low-dose protocol, by reducing the tube current to 100 mAs [6], this 128-slice CT scan has an edge over the lower-slice models without affecting the image quality for reporting. The pulmonary CTA plays an important role in TOF preparative evaluation and workup and assists by minimizing routine invasive digital subtraction catheter angiography which takes a conventional time delay of 15–17 seconds and the pulmonary CTA in less than 10 seconds [7]. The pulmonary CTA is also advantageous to lower slice scanners for follow-up and monitoring, including volumetric quantification, as well as for diagnosing pulmonary embolism, hypertension, and additional pathologies of the chest [8], and can be used in cases in which magnetic resonance imaging is contraindicated or limited due to noncompatible implanted devices like pacemakers and stents or claustrophobia.

Pulmonary CT angiography can be diagnosed by studying the complex anatomy and variations in the TOF subtypes such as absent pulmonary valve and pulmonary atresia with major aortopulmonary collateral arteries (MAPCA) [9].

Postoperatively, 128-slice CT is also very helpful in the identification of surgical complications, such as the patency of the palliative shunts such as the modified Blalock-Taussig shunt in case 3. Even these shunts lead to distortion in the anatomy which requires additional intervention [10]. Fast scan times of less than ten seconds, simultaneous image acquisition and better resolution, and fewer motion artifacts in the 128-slice CT scanner make it superior and as par as conventional imaging techniques for TOF patients and are seen as par as invasive DSA techniques. The additional advantage of conventional echocardiography is that, as in cases 2 and 3, aortopulmonary collaterals can be seen. In case 4, the better-visualized anatomy of the bilateral innominate and left-sided superior vena cava and its course in the chest. Pulmonary tuberculosis is seen in case 3 and cannot be seen by echocardiography. The mortality benefit is proven for pulmonary CTA by early diagnosis and management, making it a superior investigation technique in spite of radiation exposure [11]. Compared to the 64-slice CT scanner, the larger coverage and shorter time scan make 128-slice CT better. This also has acceptable images in low tube current and thus also minimizes dose, as seen in this study [12].

However, still, due to contrast sensitivity, deranged renal function tests, and uncooperative patients with motion, there are a few disadvantages where this technique cannot be performed. The use of general anaesthesia or sedation can solve the problem of motion in infants and uncooperative children. Deranged renal function can be a contraindication for all types of pulmonary angiography due to the use of contrast, including DSA.

## 3. Conclusion

Pulmonary CTA in the 128-slice CT scanner is very useful as it provides fast, accurate, objective information and images with less operator skill dependency with better spatial resolution and image anatomy as compared to conventional imaging and lower slice models of CT scanners. This noninvasive imaging technique is very useful for operating cardiothoracic surgeons in the case of TOF. It gives additional information with conventional echocardiography for surgical correction. The patency of shunts along with complex cardiac and extracardiac anomalies is also easily diagnosed.
